# North Star Ambulatory Assessment changes in ambulant Duchenne boys amenable to skip exons 44, 45, 51, and 53: A 3 year follow up

**DOI:** 10.1371/journal.pone.0253882

**Published:** 2021-06-25

**Authors:** Giorgia Coratti, Marika Pane, Claudia Brogna, Valeria Ricotti, Sonia Messina, Adele D’Amico, Claudio Bruno, Gianluca Vita, Angela Berardinelli, Elena Mazzone, Francesca Magri, Federica Ricci, Tiziana Mongini, Roberta Battini, Luca Bello, Elena Pegoraro, Giovanni Baranello, Stefano C. Previtali, Luisa Politano, Giacomo P. Comi, Valeria A. Sansone, Alice Donati, Jean Yves Hogrel, Volker Straub, Silvana De Lucia, Erik Niks, Laurent Servais, Imelda De Groot, Mary Chesshyre, Enrico Bertini, Nathalie Goemans, Francesco Muntoni, Eugenio Mercuri, on behalf on the International DMD Group and the iMDEX Consortium

**Affiliations:** 1 Pediatric Neurology, Department of Woman and Child Health and Public Health, Child Health Area, Università Cattolica del Sacro Cuore, Rome, Italy; 2 Centro Clinico Nemo, Fondazione Policlinico Universitario Agostino Gemelli IRCCS, Rome, Italy; 3 Dubowitz Neuromuscular Centre, UCL & Great Ormond Street Hospital, London, United Kingdom; 4 NIHR Great Ormond Street Hospital Biomedical Research Centre, London, United Kingdom; 5 Department of Clinical and Experimental Medicine, University of Messina, Messina, Italy; 6 Nemo SUD Clinical Centre, University Hospital “G. Martino”, Messina, Italy; 7 Department of Neurosciences, Unit of Neuromuscular and Neurodegenerative Disorders, Bambino Gesù Children’s Hospital, Rome, Italy; 8 Center of Translational and Experimental Myology, IRCCS Istituto Giannina Gaslini, Genoa, Italy; 9 IRCCS Mondino Foundation, Pavia, Italy; 10 Fondazione IRCCS Ca’ Granda Ospedale Maggiore Policlinico, Dino Ferrari Centre, Department of Pathophysiology and Transplantation, University of Milan, Milan, Italy; 11 Neuromuscular Center, AOU Città della Salute e della Scienza, University of Turin, Torino, Italy; 12 Department of Developmental Neuroscience, Stella Maris Institute, Pisa, Italy; 13 Department of Clinical and Experimental Medicine, University of Pisa, Pisa, Italy; 14 Department of Neurosciences, University of Padua, Padua, Italy; 15 Fondazione IRCCS Istituto Neurologico Carlo Besta, Milan, Italy; 16 Division of Neuroscience, IRCCS San Raffaele Scientific Institute, Milan, Italy; 17 Cardiomyology and Medical Genetics, University of Campania Luigi Vanvitelli, Naples, Italy; 18 The NEMO Center in Milan, Neurorehabilitation Unit, ASST Niguarda Hospital, University of Milan, Milan, Italy; 19 Metabolic Unit, A. Meyer Children’s Hospital, Florence, Italy; 20 Institute I-Motion, Hôpital Armand Trousseau, Institute of Myology, Paris, France; 21 John Walton Muscular Dystrophy Research Centre, Translational and Clinical Research Institute, Newcastle University and Newcastle Hospitals NHS Foundation Trust, Newcastle upon Tyne, United Kingdom; 22 Department of Neurology, Leiden University Medical Center, Leiden, The Netherlands; 23 Centre de Référence Des Maladies Neuromusculaires, CHU de Liège, Liège, Belgium; 24 Department of Paediatrics, MDUK Neuromuscular Center, University of Oxford, Oxford, United Kingdom; 25 Department of Rehabilitation, Donders Center for Medical Neuroscience, Radboud University Medical Center, Nijmegen, The Netherlands; 26 Department of Child Neurology, University Hospitals Leuven, Leuven, Belgium; University of Minnesota Medical School, UNITED STATES

## Abstract

**Introduction:**

The aim of this study was to report 36-month longitudinal changes using the North Star Ambulatory Assessment (NSAA) in ambulant patients affected by Duchenne muscular dystrophy amenable to skip exons 44, 45, 51 or 53.

**Materials and methods:**

We included 101 patients, 34 had deletions amenable to skip exon 44, 25 exon 45, 19 exon 51, and 28 exon 53, not recruited in any ongoing clinical trials. Five patients were counted to skip exon 51 and 53 since they had a single deletion of exon 52.

**Results:**

The difference between subgroups (skip 44, 45, 51 and 53) was significant at 12 (p = 0.043), 24 (p = 0.005) and 36 months (*p≤0*.*001*).

**Discussion:**

Mutations amenable to skip exons 53 and 51 had lower baseline values and more negative changes than the other subgroups while those amenable to skip exon 44 had higher scores both at baseline and at follow up.

**Conclusion:**

Our results confirm different progression of disease in subgroups of patients with deletions amenable to skip different exons. This information is relevant as current long term clinical trials are using the NSAA in these subgroups of mutations.

## Introduction

The North Star Ambulatory Assessment (NSAA) is increasingly used as a primary or secondary outcome measure in clinical trials in Duchenne muscular dystrophy (DMD), as it combines a reliable assessment of several aspects of motor function with a structured validated format [1, 2]. Several studies have reported longitudinal natural history data using the NSAA [3–7]. The advent of dystrophin restoration therapies targeting specific subgroups of mutations such as deletions amenable to skip individual exons using antisense oligonucleotides, or non-sense mutations using read-through small molecules strategies, has highlighted the need for establishing whether the changes in these subgroups of patients follow the mean changes observed in the whole group of DMD [4, 8–12]. A few studies have reported that there are some differences in theses subgroups both in terms of loss of ambulation and in rate of decline over time [8, 9]. Others have specifically investigated 12- and 24- month NSAA changes according to genotypes showing that some differences among subgroups can already be noted in the first year but become statistically significant in the second year [4, 10]. A longer follow up is needed as the results of exon skipping clinical trials have highlighted that efficacy is better appreciated after the first year of treatment and becomes more evident with increasing time [11, 12]. The results of long-term studies can only be interpreted using external controls matched for inclusion criteria, including genotype, as a long-term placebo arm would not be acceptable. External controls have already been used in trials with the 6-Minute Walk Test (6MWT) as the primary outcome measure using the longitudinal data available in the literature [11, 12]. Given the increasing number of ongoing and planned studies targeting deletions amenable to skip individual exons (NCT03218995, NCT03375255, NCT02500381, NCT02310906, NCT03508947, NCT02081625), using the NSAA as the primary or secondary outcome measure, it is essential to further evaluate long-term information on the NSAA patterns of progression in relation to the different genotypes.

The aim of our collaborative effort was to obtain longitudinal prospective changes over 36 months in the NSAA in a large cohort of DMD patients amenable to skip exons 44, 45, 51 and 53. We also aimed to establish if within each genetic subgroup, age, Time to Raise from Floor (TRF) or baseline NSAA could help to identify patients at risk of loss of ambulation or reduced ambulatory activity.

## Materials and methods

The study is part of a longitudinal multi-centric cohort effort involving 12 tertiary neuromuscular centers in Italy, two centers in the United Kingdom, one in France, one in the Netherlands and one in Belgium. Patients were recruited between January 2008 and September 2017 and followed for at least three years. Inclusion criteria at baseline were: genetically confirmed DMD diagnosis, patient still ambulant and able to walk independently for at least 150 meters at baseline, no severe or moderate intellectual disability or behavioral problems.

In this manuscript, only patients with deletions amenable to skip exons 44, 45, 51 and 53 and who are not taking part in interventional trials/receive commercially available drugs were included. Each center included in the project had approval from the local Ethical Committee (Catholic University, Rome, Ospedale Bambino Gesù, Rome; Istituto Mondino, Pavia; Gaslini Institute, Genova; Besta Institute, Milan; Stella Maris Institute, Pisa; University of Napoli, University of Messina; AOU Città della Salute e della Scienza, Turin; University of Padova; University of Milano; Centro Clinico Nemo Milano, University Hospitals Leuven, Belgium; NHS Multicentric Research Ethics Committee, England; CPP-Ile-de-France VI, Paris, France).

The informed consent signed by parents of participants and patients included in this study declared that all data relevant to the disease (e.g. steroids, functional assessments) and collected as part of clinical routine could be included, in an anonymous format, in observational studies defining natural history.

### North Star Ambulatory Assessment (NSAA)

The NSAA is an ordinal scale consisting of 17 items, ranging from standing to running. It includes several items assessing abilities such as head raise and standing on heels and a number of dynamic activities such as hopping or running [2]. Each item can be scored on a 3-point scale: 2 –Normal achieves goal without any assistance; 1 –Modified method but achieves goal independent of physical assistance from another person; 0 –Unable to achieve independently. The score ranges from 0, if all the activities are failed, to 34, if all the activities are achieved.

As part of the clinical routine, patients were seen at least every 12 months. Data were collected from recruitment (baseline) and at 12, 24, 36-months after. Details on functional assessment training and of the inter-observer reliability among physiotherapists have already been reported [1, 3].

### Time to rise from the floor (TRF)

As part of this study, all boys were asked to perform the timed items, including time to rise from the floor (TRF). The TRF was performed recording the time (seconds) taken to complete the task of rising from supine to full standing [13].

The boys who were able to perform the task were subdivided into two groups, based on the ability to perform the task in less or more than 6 seconds as used in previous studies [14]. Boys that were unable to complete the task were categorized together with those able to complete the task in more than 6 seconds.

### Statistical analysis

The NSAA was evaluated longitudinally at 12, 24 and 36 months after baseline. Descriptive statistics (N, mean, SD, range) were used. Descriptive analysis also included assessment of patients who lost ambulation (defined an inability to walk 10 meters independently) or who reached a NSAA score <6, associated with limited functional ability.

Mann–Whitney *U* test was used to compare differences between groups on the NSAA at baseline and at 12, 24, 36-month assessments or their changes from baseline. The test was performed according to age subgroups (<7, ≥7 years), TRF at baseline (<6, ≥6 sec) or NSAA level at baseline (≤22, >22 score). Chi-square test was used to analyze TRF (<6, ≥6 sec) distribution among the skipping subgroup (44, 45, 51, 53).

Analysis of variance model (ANOVA) with Bonferroni correction was used to assess heterogeneity among groups of the NSAA at baseline and at 12, 24, 36-month assessments or their changes from baseline. The test was performed according to the type of skipping exons 44, 45, 51, 53 and the steroids regimen at baseline (naive vs alternate or continuous regime). For all the analysis the p-value was set at < 0.05.

## Results

Of the 131 DMD patients who had deletions amenable to skip exons 44, 45, 51 and 53, 101 (mean age 7.7 years; SD±2.3) fulfilled the inclusion criteria and entered the study. The remaining 30 were not recruited either because of participation in clinical trials (n = 22), because they had been followed for less than three years (n = 4), or were lost at follow up (n = 4).

Thirty-four of the 101 had deletions amenable to skip for exon 44, 25 amenable to skip for exon 45, 19 had deletions amenable to skip for 51, and 28 had deletions amenable to skip exon 53. Of the patients with a single deletion of exon 52, five were counted in both subgroups amenable to skip exon 51 and 53. Of the 101 boys, 43 were under 7 years of age at the time of the first assessment (11 not on steroids, 12 on alternate and 20 on continuous steroids), 58 were above or equal to 7 years (5 not on steroids, 26 on alternate and 27 on continuous steroids). None of the patients discontinued steroid treatment during the study.

### North Star Ambulatory Assessment (NSAA)

A difference on NSAA scores was found between the groups under and above 7 years of age at 12 months (p = 0.001), at 24 months (p<0.001) and at 36 months (p<0.001), with patients ≥7 years of age at baseline having lower NSAA mean scores; no statistical difference was found comparing baseline values (Table 1).

**Table 1:** Details of the NSAA in the study cohort and subdivided by age at baseline (<7 or ≥7 years).

Group	N	Age at baseline, mean (SD)	NSAA at baseline, mean (SD)	NSAA at 12 months, mean (SD)	NSAA at 24 months, mean (SD)	NSAA at 36 months, mean (SD)
**All deletions amenable to skip 44, 45, 51 and 53**	101	7.7 (±2.3)	24.1 (±6.5)	22.9 (±8.2)	20.2 (±9.8)	17.5 (±10.9)
**Amenable to exon skipping <7 years**	43	5.5 (±0.8)	24.6 (±5.2)	26.1 (±5.5)	25.0 (±6.2)	23.5 (±8.4)
**Amenable to exon skipping ≤7 years**	58	9.3 (±1.6)	23.7 (±7.4)	20.5 (±9.0)	16.3 (±10.5)	13.1 (±10.5)
**Statistical significance between <7 and ≥ 7years (p)**			p = 0.47	p = 0.001	p<0.001	p<0.001

A difference on NSAA scores was also found between the groups under and above a baseline score of 22 on the NSAA, at 12 months (p<0.001), at 24 months (p<0.001) and at 36 months (p< 0.001), with patients performing a score ≤22 having lower NSAA mean scores.

Of the 101 patients, 21 lost ambulation within 36 months from baseline. The mean age at loss of ambulation was 11.4 years (SD±1.9).

In the group of patients < 7 years of age at baseline, a difference on the NSAA scores was found among the different steroids’ groups (no steroids vs alternate vs continuous regime) at baseline (p = 0.040) and at 36 months assessments (p = 0.014) but not at 12- or 24-months assessments. Post hoc comparisons using the Bonferroni correction indicated that, at baseline, the mean score for the steroid’s naïve patients (Mean = 21.6, SD = ±5.9) was significantly lower than the patients doing daily steroid regime (Mean = 26.4, SD = ±3.6) (p = 0.039). At 36 months, the mean scores for the alternate steroid regime (Mean = 17.6, SD = ±9.9) was significantly lower than the mean scores for the daily steroid regime (Mean = 25.3, SD = ±7.1) (p = 0.025).

In the group of patients ≥ 7 years of age at baseline, no difference on the NSAA was found among the different steroids’ regimen groups (no steroids vs alternate vs continuous regimen) at baseline, 12 or 24 months or at 36 months assessments.

#### Time to rise from the floor (TRF)

Sixty-six of the 101 patients had TRF below 6 sec and 32 had TRF above or equal to 6 seconds at baseline. No data were available from 3 patients (1 amenable to skip for exon 51, 2 exon 44 and 45).

There was a difference on both NSAA scores and NSAA changes in patients below and above 6 seconds TRF at baseline (*p*≤0.001), 12 months (*p*≤0.001), 24 months (*p*≤0.001) and at 36 months (*p*≤0.001), with patients above 6 seconds having lower mean scores and higher mean changes on the NSAA (Table 2).

**Table 2:** Details of the NSAA in the study cohort subdivided by TRF at baseline (<6 or ≥6 years).

		North Star Ambulatory Assessment						
TRF		Baseline	12 months	24 months	36 months	12-month change	24-month change	34-month change
**< 6 SECONDS (n:66)**	*Mean*	27	27.3	25.3	23.2	-0.1	-1.9	-3.8
	*SD*	±4.4	±4.5	±5.6	±7.8	±5.3	±7.0	±9.0
	*Min*	13	13	10	0	-24	-31	-30
	*Max*	34	34	33	33	13	14	14
**≥ 6 SECONDS (n:32)**	*Mean*	18.5	14.1	9.4	6.1	-4.8	-9.7	-12.4
	*SD*	±5.9	±6.1	±7.4	±6.6	±4.6	±7.4	±6.6
	*Min*	4	0	0	0	-13	-27	-26
	*Max*	31	28	29	27	6	10	8
**Statistical significance between <6 and ≥ 6 seconds (p)**		*p*≤0.001	*p*≤0.001	*p*≤0.001	*p*≤0.001	*p*≤0.001	*p*≤0.001	*p*≤0.001

In the group with baseline TRF ≥6 sec, the NSAA scores at baseline ranged between 4 and 31 (Mean = 18.5, SD = ±5.9) and 24 of the 32 (75%) had scores ≤22. In the group with baseline TRF <6 sec, the NSAA scores at baseline ranged between 13 and 34 (Mean = 27, SD = ±4.4) and 9 of the 66 (13%) had scores ≤22.

#### Patients amenable to skip exon 44 (n = 34)

**Fig 1:**
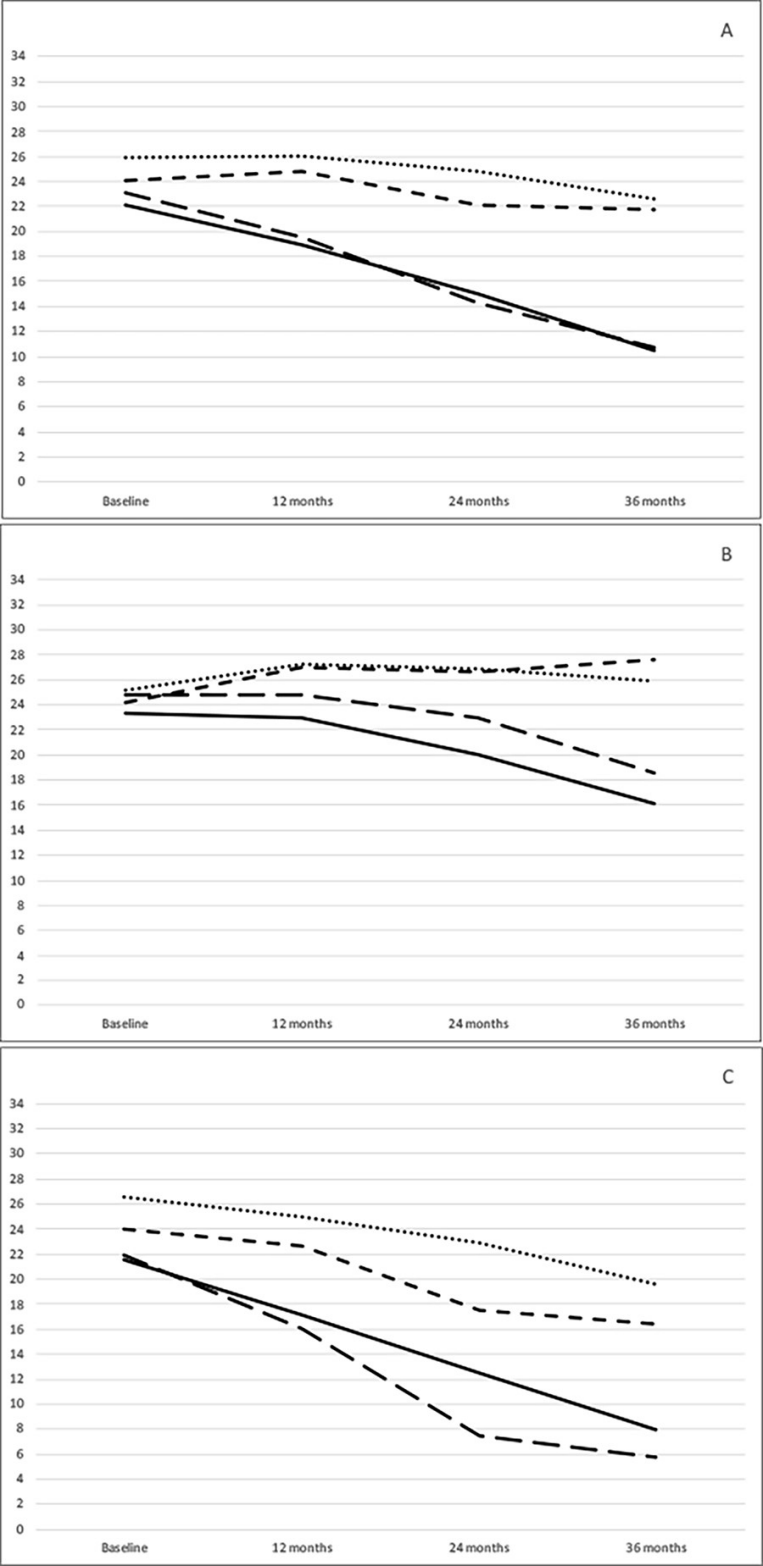
Mean NSAA changes in the subgroups amenable to skip exons 44, 45, 51 and 53 according to age. Panel A: Study Cohort. Panel B: <7 years of age. Panel C: ≥7 years of age. ··· = amenable to skip 44, — = amenable to skip 45,––– = amenable to skip 51,––– = amenable to skip 53.

**Fig 2:**
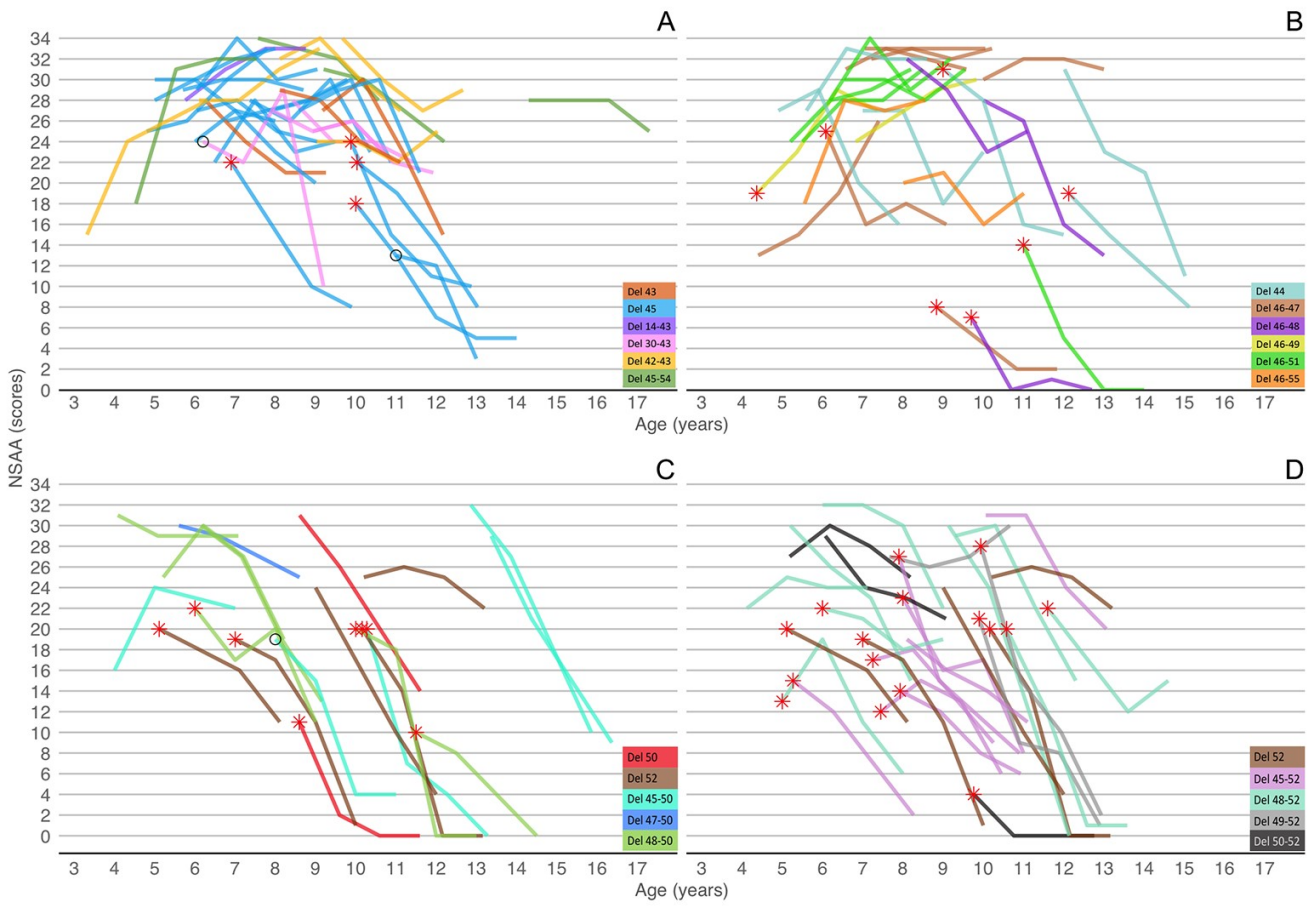
Individual NSAA trajectories with details of the mutations within skipping subgroups. Panel A: Cohort amenable to skip exon 44 (n = 34). Panel B: Cohort amenable to skip exon 45 (n = 25). Panel C: Cohort amenable to skip exon 51(n = 19). Panel D: Cohort amenable to skip exon 53 (n = 28). * = TRF ≥ 6 sec, ○ = TRF not performed.

The mean NSAA was 25.9 at baseline. Mean changes from baseline were +0.1 at 12 month, -1.8 mt at 24 months and– 3.3 at 36 months. Sixteen were younger than 7 years of age and 18 were above or equal to 7 years (Table 3; Figs 1 and 2).

**Table 3:** Baseline 12-, 24-, 36-month NSAA values (range, mean and median) subdivided according to genotype and age.

	Skip group		Age	NSAA BASELINE	NSAA 12 MONTHS	NSAA 24 MONTHS	NSAA 36 MONTHS	CHANGES 12-MONTH	CHANGES 24-MONTH	CHANGES 36-MONTH
**All**	*44 (N*:*34)*	*Mean (SD)*	7.5 (+2.2)	25.9 (+4.8)	26.0 (+6.0)	24.8 (+7.1)	22.6 (+8.4)	0.1 (+4.3)	-1.8 (+7.7)	-3.3 (+7.3)
		*Min; max*	3.3; 14.3	13; 34	7; 34	5; 33	3; 33	-9; 13	-31; 14	-15; 14
	*45 (N*:*25)*	*Mean (SD)*	7.5 (+2.3)	24.1 (+7.5)	24.7 (+9.1)	22.1 (+10)	21.8 (+11)	-1.3 (+6.9)	-2.9 (+7.9)	-2.3 (+9)
		*Min; max*	4.4; 12.1	7; 33	0; 34	0; 33	0; 33	-24; 10	-19; 10	-20; 13
	*51 (N*:*19)*	*Mean (SD)*	8.2 (+2.8)	22.8 (+6.6)	19.4 (+8.2)	14.1 (+10.5)	10.2 (+9.5)	-3.5 (+5.0)	-7.9 (+7.8)	-12.6 (+7.9)
		*Min; max*	4; 13.4	10; 32	2; 30	0; 29	0; 29	-13; 8	-20; 7	-22; 6
	*53 (N*:*28)*	*Mean (SD)*	7.8 (+2.1)	22.2 (+6.7)	19.0 (+7.5)	15.1 (+8.7)	10.9 (+9.1)	-3.2 (+4.9)	-7.8 (+7.4)	-11.4 (+8.9)
		*Min; max*	4.1; 11.6	4; 32	0; 32	0; 30	0; 30	-13; 6	-27; 2	-30; 3
**<7 years**	*44 (N*:*16)*	*Mean (SD)*	5.7 (+0.9)	25.2 (+4.2)	27.2 (+4.3)	26.9 (+5.7)	25.9 (+7.9)	2.1 (+4.6)	1.7 (+6.1)	0.75 (+8.0)
		*Min; max*	3.3; 6.9	15; 30	16; 34	10; 33	8; 33	-6; 13	-12; 14	-14; 14
	*45 (N*:*12)*	*Mean (SD)*	5.6 (+0.8)	24.2 (+5.2)	27 (+6.5)	26.7 (+4.9)	27.6 (+5.7)	0.6 (+9)	2.5 (+5.5)	3.43 (+7.2)
		*Min; max*	4.4; 6.8	13; 31	15; 34	18; 32	16; 32	-24; 10	-7; 10	-11; 13
	*51 (N*:*7)*	*Mean (SD)*	5.2 (+0.9)	24.9 (+58)	24.9 (+5.4)	23 (+4.9)	18.6 (+7.2)	0 (+4.7)	-1.9 (+5.5)	-6.3 (+7.2)
		*Min; max*	4; 6.2	16; 31	17; 30	16; 29	11; 29	-5; 8	-11; 7	-17; 6
	*53 (N*:*9)*	*Mean (SD)*	5.3 (+0.6)	23.3 (+6.7)	23 (+6.2)	20 (+7.6)	16.1 (+8.2)	-0.3 (+3.7)	-3.3 (+3.4)	-7.2 (+5.4)
		*Min; max*	4.1; 6.1	13; 32	12; 32	7; 30	2; 25	-5; 6	-8; 2	-15; 2
**≥7 years**	*44 (N*:*18)*	*Mean (SD)*	9.2 (+1.6)	26.6 (+5.3)	24.9 (+7.2)	22.9 (+7.9)	19.6 (+7.9)	-1.6 (+3.1)	-4.9 (+7.7)	-7 (+4.2)
		*Min; max*	7.4; 14.3	13; 34	7; 34	5; 32	3; 29	-9; 3	-31; 3	-15; 1
	*45 (N*:*13)*	*Mean (SD)*	9.2 (+1.8)	24 (+9.4)	22.7 (+10.8)	17.5 (+11.8)	16.4 (+12.1)	-3.1 (+3.9)	-7.8 (+6.3)	-7.6 (+7.0)
		*Min; max*	7; 12.1	7; 33	0; 33	0; 33	0; 33	-9; 2	-19; 2	-20; 1
	*51 (N*:*12)*	*Mean (SD)*	9.9 (+1.9)	21.7 (+6.9)	16.2 (+8.0)	7.8 (+8.7)	5.3 (+7.1)	-5.5 (+4.1)	-12.1 (+6.3)	-16.3 (+5.6)
		*Min; max*	8; 13.4	10; 32	2; 27	0; 25	0; 22	-13; 1	-20; 0	-22; -3
	*53 (N*:*19)*	*Mean (SD)*	9.0 (+1.3)	21.7 (+6.8)	17.2 (+7.5)	12.6 (+8.3)	8.4 (+8.6)	-4.5 (+5)	-10.1 (+7.9)	-13.3 (+8.5)
		*Min; max*	7; 11.6	4; 31	0; 31	0; 27	0; 30	-13; 3	-27; 1	-30; 3

Two patients lost ambulation in the third year, with a mean age at loss of ambulation of 13.5 years (SD ±0.7). TRF at baseline was ≥6 sec in one and unknown in the other. TRF at baseline ≥6 sec was also present in 3/32 (9%) who did not lose ambulation.

A difference in the NSAA scores was found when subdividing the groups into below and above 6 seconds TRF at baseline (*p* = 0.005), 12 months (*p*≤0.001), 24 months (*p*≤0.001) and 36 months (*p*≤0.001), with patients above 6 seconds having lower mean scores in the NSAA assessment.

A difference was also found in the NSAA changes at 12-month (*p*≤0.001), 24-month (*p*≤0.002), and 36-month (*p*≤0.001) changes, with patients above 6 seconds having higher mean changes scores.

In the group of patients ≥ 7 years of age at baseline, no difference in the NSAA was found among the different steroids’ groups (no steroids vs alternate vs continuous regime) at baseline, 12, 24, 36 months or their changes, while in the group of patients <7 years of age at baseline, a trend was observed at 12-month changes (p = 0.036) and 36-month changes (p = 0.037), but this result was not significant after Bonferroni correction.

#### Patients amenable to skip exon 45 (n = 25)

The mean NSAA was 24.1 at baseline. Mean changes from baseline were -1.3 at 12 months, -2.9 at 24 months and -2.3 mt at 36 months. Twelve were younger than 7 years of age and 13 were above or equal to 7 years (Table 3; Figs 1 and 2).

Three lost ambulation during the study, two at 12- and one at 24- from baseline, with a mean age at loss of ambulation of 11 years (SD ±1.7). All three (100%) had a TRF ≥6 sec at baseline. TRF at baseline ≥6 sec was also present in 4/22 (18%) who did not lose ambulation.

A difference in the NSAA scores was found when subdividing the groups into below and above 6 seconds TRF at baseline (*p* = 0.021), 12 months (*p* = 0.002), 24 months (*p* = 0.007), at 36 months (*p* = 0.001), with patients above 6 seconds having lower mean scores in the NSAA assessment.

A difference was also found in the NSAA changes at 12-month (*p* = 0.017) but not at 24-month (p = 0.64) nor at 36-month follow up (p = 0.64), with patients above 6 seconds having higher mean changes scores. No difference in the NSAA was found among the different steroids’ regimen groups (no steroids vs alternate vs continuous regimen) at baseline, 12, 24, 36 months or their changes, irrespective of age at baseline (<7 or ≥7 years).

#### Patients amenable to skip exon 51 (n = 19)

The mean NSAA was 22.8 at baseline. Mean changes from baseline were -3.5 at 12 months, -7.9 mt at 24 months and -12.6 at 36 months. Seven were younger than 7 years of age and 12 were above or equal to 7 years (Table 3; Figs 1 and 2).

Seven lost ambulation during the study, one at 12-, five at 24- and one at 36 months from baseline, their mean age at loss of ambulation was 11.4 years (SD ±1.8). Six had a TRF at baseline ≥6 sec at baseline and in the remaining one TRF at baseline was unknown. TRF at baseline ≥6 sec was also present in 2/13 (15%) who did not lose ambulation.

A difference on the NSAA scores was found when subdividing the groups into below and above 6 seconds TRF at baseline (*p* = 0.001), 12 months (*p* = 0.001), 24 months (*p* = 0.002), at 36 months (*p* = 0.001), with patients above 6 seconds having lower mean scores on the NSAA assessment.

A difference on the NSAA scores was not found when subdividing the groups into below and above 6 seconds TRF at 12, 24 or 36-month changes (*p*>0.05). No difference on the NSAA was found among the different steroids’ groups (no steroids vs alternate vs continuous regime) at baseline, 12, 24, 36 months or their changes, irrespective of age at baseline (<7 or ≥7 years).

#### Patients amenable to skip exon 53 (n = 28)

The mean NSAA was of 22.2 at baseline. Mean changes from baseline were of -3.2 mt at 12 months, -7.8 at 24 months and -11.4 at 36 months.

Nine were younger than 7 years of age and 19 were above or equal to 7 years (Table 3; Figs 1 and 2). Nine lost ambulation during the study, four at 24- and five at 36 months from baseline, their mean age at loss of ambulation was 11 years (SD ±2).

Eight had a TRF at baseline ≥6 sec and in the remaining one TRF at baseline was <6 seconds (4.4 seconds). TRF at baseline ≥6 sec was also present in 8/19 (42%) who did not lose ambulation.

A difference on the NSAA scores was found when subdividing the groups into below and above 6 seconds TRF at baseline (*p*≤0.001), 12 months (*p*≤0.001), 24 months (*p*≤0.001) at 36 months (*p* = 0.001), with patients above 6 seconds having the lowest mean scores to the NSAA assessment.

A difference on the NSAA scores was not found when subdividing the groups into below and above 6 seconds TRF at 12, 24 or 36-month changes (*p*>0.05). No difference on the NSAA was found among the different steroids’ groups (no steroids vs alternate vs continuous regime) at baseline, 12, 24, 36 months or their changes, irrespective of age at baseline (<7 or ≥7 years).

### NSAA changes from baseline subdivided by skip group

The proportion of subjects who had a TRF <6 or ≥6 at baseline differ by skip group (X^2^(3 = 14.55) p = 0.002), with 12% and 38% of patients amenable to skip 44 and 45 respectively having a TRF ≥6 seconds at baseline compared to 44% and 57% of patients amenable to skip 51 and 53 respectively. TRF ≥6 seconds was reached at a mean age of 9.2 years (SD ±1.5) in patients amenable to skip exon 44, at 8.7 years (SD ±2.7) in patients amenable to skip exon 45, at 8.6 years (SD ±2.3) in patients amenable to skip exon 51 and at 8.0 years (SD ±2.1) in patients amenable to skip exon 53. The difference among individual subgroups was only significant between those amenable to skip exon 44 and 53.

When we analyzed the NSAA mean changes from baseline, there was a significant difference between deletions amenable to skip exons 44, 45, 51 or 53 at 12 (p = 0.043), 24 (p = 0.005) and 36 months (*p≤0*.*001*). Post hoc comparisons using the Bonferroni correction indicated that at 12 months there was no significant difference among individual subgroups. At 24 months, there was a difference only between patients amenable to skip exon 44 (Mean = -1.8, SD = ±7.7) and patients amenable to skip exon 53 (Mean = -7.8, SD = ±7.3) (p = 0.016). At 36 months, there was a difference between patients amenable to skip exon 44 (Mean = -3.3, SD = ±7.3) and both patients amenable to skip exon 51 (Mean = -12.3, SD = ±7.9) (p = 0.001) and exon 53 (Mean = -11.6, SD = ±8.0) (p = 0.001). There was also a difference between patients amenable to skip exon 45 (Mean = -2.3, SD = ±8.9) and those amenable to skip exon 51 (p = 0.001) or exon 53 (Mean = -11.6, SD = ±8.0) (p<0.001). There was no difference between patients amenable to skip exon 44 (Mean = -3.3, SD = ±7.3) and 45 (Mean = -2.3, SD = ±8.9) or between patients amenable to skip exon 51 (Mean = -12.3, SD = ±7.9) and 53 (Mean = -11.6, SD = ±8.0).

## Discussion

The results of our study assessing 36-month NSAA changes in DMD patients amenable to skip exons 44, 45, 51 and 53 confirm the trends observed using the 6MWT [14]. In general, the subgroups amenable to skip 44 and 45 had better baseline scores and the decline in scores occurred at a later age when compared to those amenable to skip exon 51 and 53. Patients amenable to skip 51 and 53 showed a decrease in mean NSAA scores already below the age of 7 years, while this did not occur in those amenable to skip exons 44 and 45. The decline before the age of 7 years of the subgroups amenable to skip 51 and 53 was not observed in previous studies exploring 3 years NSAA changes in whole cohorts of DMD, or in groups including all deletions, all duplications or small mutations [15]. After the age of 7 years, all the subgroups in our study showed some decline, but the magnitude of changes was different as the negative changes in patients amenable to skip exon 44 or 45 (-7 points) were smaller than those observed in patients for exons 51 and 53 (-16 and -13 respectively).

The different decline was also supported by the observation that in patients with mutations amenable to skip exons 44 and 45, loss of ambulation occurred less frequently (44 = 6%, 45 = 12%) than in those amenable to skip exon 51 and 53 (37% and 32%). More generally, NSAA scores lower than 6, associated with very restricted functional ability were found in many patients amenable to skip exon 53 and 51 (32% and 42% respectively), and were often observed already soon after the age of 8–9 years while such low scores occurred only in two patients amenable to skip exon 44 after the age of 12 years and in three patients amenable to skip exon 45 between the age of 9 and 12 years.

These results are consistent with other studies investigating differences between patients eligible to skip different exons. In particular, patients eligible to skip exon 44 are reported to have higher level of revertant fibers and traces of dystrophin expression than those eligible to skip exon 51 or 53 [16]. This has been reported to be related to the fact that exon 44 may skip spontaneously when surrounding exons are deleted [17].

The difference among subgroups did not appear to be related to steroid regime but the interpretation of these results is limited by the fact that when identifying different regimes in the small subgroups, the numbers were very small.

In this paper we were also interested to establish clinical features that may help to predict functional decline in the different subgroups. In agreement with previous findings reporting that TRF can be used to predict functional decline and loss of ambulation [13, 18], we confirmed that, TRF≥6 seconds were more often associated with loss of ambulation and higher mean negative changes when compared to patients with TRF< 6sec (85% vs 4%).

This occurred in all genetic subgroups but the age when patients reached the point of TRF decline was different, as patients amenable to skip 51 and 53 reached TRF>6 sec more often and at an earlier age compared to those amenable to skip 45 and 44. Interestingly, most patients (83%) with TRF ≥6 sec also had NSAA scores ≤22 that had previously been reported to be associated with increased risk of loss of ambulation within 2 years.

Our study, using the NSAA, confirmed that subgroups of DMD patients amenable to skip different exons have a different progression over 3 years, as previously reported in a study assessing the 6MWT changes.

Unfortunately, the cohort in the present study was not completely overlapping with the cohort reported in the 3-year 6MWT study [14], as in some centers only one of the two functional assessments was performed. The two cohorts cannot be matched and easily compared, but both studies strongly demonstrate that patients amenable to skip exon 51 and 53 have a faster decline and have an increased risk of losing ambulation over 36-month follow up. Even if the numbers in each subgroup were relatively small, these results provide some reference data illustrating the long term NSAA changes in each subgroup. These results will be useful for designing clinical trials targeting deletions amenable to skip individual exons providing a better knowledge on the expected results in the untreated placebo group. Furthermore, as we provide long term results, our findings will also be of help for the interpretation of long-term real-world data or of the results of ongoing extension trials in which a placebo arm is either not used or limited to the first 12–18 months.
